# Biofilm, virulence, and ESBL-mediated resistance: unmasking *Klebsiella* spp. UTIs

**DOI:** 10.1128/spectrum.00664-26

**Published:** 2026-07-27

**Authors:** Maisha Samio, Bushra Benta Rahman Prapti, Kazi Mohimen Alam Arnop, Md. Tanjir Ahmmed, Aminur Rahman, Md. Al Amin, Mohammad Afzalur Rahman, Nazia Haque, Sabina Yasmin, Mahbubul Pratik Siddique

**Affiliations:** 1 Department of Microbiology and Hygiene, Faculty of Veterinary Science, Bangladesh Agricultural University 54492, Mymensingh, Bangladesh; 2 ICDDRB: International Centre for Diarrhoeal Disease Research 56291 , Dhaka, Bangladesh; 3 Mymensingh Medical Collage, Mymensingh, Bangladesh; 4 Institute of Biotechnology, Bangladesh Agricultural University 54492, Mymensingh, Bangladesh; Universidad Maimonides, Buenos Aires, Argentina

**Keywords:** *Klebsiella *spp., urinary tract infection (UTI), MDR, virulence genes, biofilm, ESBL and carbapenemase genes

## Abstract

*Klebsiella* spp. has emerged as the second leading cause of urinary tract infection and a major cause of multidrug resistance. This study investigated the prevalence of genetic determinants related to infection and drug resistance, and the effects of biofilm on drug resistance from urinary tract infection (UTI) patients. A total of 718 clean voided midstream urine samples were collected from different age group patients at Mymensingh Medical College Hospital. Bacterial isolates were initially identified by cultural and biochemical assays, and finally confirmed by *rcs*A and *phe*X gene-based polymerase chain reaction (PCR). Antimicrobial susceptibility testing was performed by the disc diffusion method. The presence of virulence, extended-spectrum beta-lactamase (ESBL), and carbapenemase genes was determined by PCR. Biofilm formation was assessed using Congo red agar and microtiter plate assays. Among culture-positive isolates, 22 were confirmed as *Klebsiella* spp. (20 *K. pneumoniae*, 2 *K. oxytoca*), with 77.3% from females. The *fim*H, *mrk*D, and *uge* were the most prevalent virulence genes. Ninety-five percent of isolates were found to be multidrug resistant (MDR), and three were extremely drug resistant (XDR). ESBL genes *bla_SHV_* (50%) and *bla_TEM_* (40%) predominated, while *bla_NDM_* (20%) and *bla_OXA-48_* (10%) were the main carbapenemase genes. Moderate (*n* = 6; 27.3%) to strong (*n* = 4; 18.1%) biofilm producers showed significantly higher resistance (*P* < 0.05). Regression analysis identified *mrk*D, *uge*, and *all*S as independent predictors of biofilm formation. The high prevalence of MDR *K. pneumoniae* with virulence and ESBL-carbapenemase genes highlights an alarming threat to UTI management in Bangladesh.

**IMPORTANCE:**

*Klebsiella* spp., particularly *K. pneumoniae* and *K. oxytoca*, are emerging as the second major uropathogens increasingly resistant to multiple antibiotics, complicating treatment and driving recurrent urinary tract infections. Their virulence factors, especially adhesins and biofilm formation, not only enhance colonization but also promote the spread of resistance genes, including extended-spectrum beta-lactamases (ESBLs) and carbapenemases. Despite their clinical significance, data on the molecular determinants of virulence and resistance in uropathogenic *Klebsiella* from Bangladesh remain limited. This study combines molecular and phenotypic approaches to characterize virulence genes, biofilm-forming potential, and antimicrobial susceptibility patterns of clinical isolates. By identifying key predictors of biofilm-associated multidrug resistance, the findings provide critical insights for guiding empirical therapy, informing infection control, and supporting antimicrobial stewardship. Ultimately, this work contributes to addressing the dual threat of virulence and drug resistance in urinary tract infection (UTI) management.

## INTRODUCTION

Urinary tract infection (UTI) represents a clinical burden, affecting approximately 150 million individuals annually worldwide and accounting for up to 35% of nosocomial infections (1, 2). Clinical manifestations range from asymptomatic bacteriuria to severe complications, such as pyelonephritis, perinephric abscess, and sepsis (3). Women are disproportionately affected due to anatomical and hormonal factors, with a lifetime incidence exceeding 50%, and occur most frequently among individuals aged 16–64 years (1). *Escherichia coli* is the predominant uropathogen, responsible for up to 75%–90% of UTI cases, while *Klebsiella* species represent the second most significant cause (2, 4). Among them, *K. pneumoniae* accounts for 86% of *Klebsiella*-related infections, making it the most significant pathogen of this genus, followed by *K. oxytoca,* which contributes to around 26% of cases (5, 6). Beyond UTIs, *Klebsiella* spp. is capable of causing respiratory tract infections, soft tissue and wound infections, liver abscesses, sepsis, and septicemia (7–9). The frequent occurrence of this pathogen, coupled with rising antimicrobial resistance, has complicated empirical therapy and increased the chance of treatment failure, recurrent infections, and prolonged hospitalization (10).

Beyond their clinical prevalence, the pathogenic potential of *Klebsiella* spp. is strongly influenced by a wide array of virulence determinants (11). A main feature of *Klebsiella* pathogenicity is the uptake of iron from the host body, which is critical for survival within the urinary tract (12). Siderophore-encoding genes, such as *ent*B (enterobactin), *iut*A (aerobactin receptor), *ybt*S, *irp*1/2, and *fyu*A (*y*ersiniabactin uptake), enhance iron uptake and are frequently associated with hypervirulence (13). The bacterial capsule and lipopolysaccharide further contribute to pathogenicity by protecting them from phagocytosis, thereby resulting in sepsis and septic shock (14). Capsular serotype K1 or K2 is particularly linked to hypervirulence, with the presence of genes, such as K1-specific gene (*mag*A) and plasmid-borne gene (*rmp*A), driving a hypermucoviscous phenotype (15). Adhesion also plays a vital role in urinary tract colonization; type 1 fimbrial adhesin (*fim*H) facilitates attachment to uroepithelial cells, and type 3 fimbrial components (*mrk*A and *mrkD*) mediate adherence to host tissue and abiotic surfaces, promoting biofilm formation (14, 15)*.* Biofilm-forming *K. pneumoniae* poses a major therapeutic challenge in UTIs, as biofilm-embedded bacteria exhibit increased tolerance to antimicrobials and host immune responses, leading to persistence and recurrent infections (14). Moreover, biofilm also serves as reservoirs of resistance genes, facilitating the emergence of multidrug resistance (MDR), extensively drug resistance (XDR), and pan-drug resistance (PDR) (16).

MDR *Klebsiella*-mediated UTIs are another major concern, which has become a significant global health issue, threatening the effectiveness of available treatments and increasing morbidity, mortality, and healthcare costs (1, 17). Moreover, inappropriate antibiotic use remains widespread in the management of CA-UTI (18). This issue is especially profound in developing countries, where many patients obtain antibiotics directly from community pharmacies without prescriptions or professional medical guidance (19). *K. pneumoniae* and *K. oxytoca*, both members of the notorious ESKAPE group, are particularly worrisome due to their ability to evade most of the available antimicrobials (5, 20). The rapid acquisition of resistance by these bacteria is largely driven by the selective pressure of inappropriate antibiotic use both in clinical and community settings, coupled with horizontal transfer of resistance genes (21).

Furthermore, the management of *K. pneumoniae* UTIs has become progressively challenging due to their diverse resistance mechanisms, which are often mediated through the production of carbapenemases and extended-spectrum β-lactamases (ESBLs) (such as KPC and NDM) and plasmid-mediated AmpC enzymes, which severely limit therapeutic options and make treatment outcomes unpredictable (22–24). These resistance genes are often carried on mobile genetic elements, including plasmids, integrons, and transposons, facilitating horizontal gene transfer between strains and species (25, 26). Through this advantage of plasmids and transferable genetic elements, *K. pneumoniae* continues to accumulate antibiotic resistance genes, driving the emergence of XDR, and often called the “super resistome” (24).

Given the clinical significance of *K. pneumoniae* and *K. oxytoca* in UTIs, understanding virulence determinants and biofilm-forming capacity is crucial. Such studies are essential for guiding empirical therapy, implementing infection control measures, and informing authorities to mitigate the impact of antimicrobial resistance. Therefore, this study aimed to investigate the virulence factors, biofilm formation potential, and antimicrobial susceptibility pattern of ESBL-producing *K. pneumoniae* and *K. oxytoca* from UTI cases. By combining molecular and phenotypic approaches, the research seeks to provide a comprehensive understanding of the dual threat posed by virulence and multidrug resistance in uropathogenic *Klebsiella* spp., contributing to informal clinical management and the broader effort to combat antimicrobial resistance.

## MATERIALS AND METHODS

### Sample collection and sampling

A total of 718 urine samples from different age groups were collected from Mymensingh Medical College Hospital during the study period. We retrospectively collected the patient data (age, sex, burning sensation during micturition, lower abdominal pain, scanty micturition, frequency of micturition, and fever) and collected clean voided midstream urine samples in sterile uricols packs (urine collection pack). All the patients enrolled in the study had not taken any antibiotics for at least 3 days before sampling.

### Assessment of UTI and isolation of *Klebsiella* spp.

Among the 718 urine samples, only 125 samples yielded 105 CFU/mL bacterial counts and were considered indicative of bacterial-driven urinary tract infections (according to the standard operating procedure, developed by the Institute of Epidemiology, Disease Control and Research, Bangladesh). Samples were further cultured on HiCrome UTI agar (Himedia, India) and incubated for 24 h at 37°C. Colonies exhibiting mucoid, blue to purple color were selected as *Klebsiella* spp. Representative colonies were further sub-cultured on MacConkey agar (Himedia, India) to enhance specificity and obtain pure cultures.

### Identification of *Klebsiella* spp.

The isolates were initially identified by Gram staining and a series of standard biochemical assays such as urease activity, indole test, and carbohydrate fermentation tests by following (27). For confirmatory detection at the species level, polymerase chain reaction (PCR) was performed targeting the *rcs*A and *peh*X genes, specific for *K. pneumoniae* and *K. oxytoca*, respectively. The target gene names, oligonucleotide sequences, expected amplicon sizes, and thermal conditions are mentioned in Table 1.

**TABLE 1:** Oligonucleotide sequence and PCR conditions of used primers

Target gene	Primer sequence (5’–3’)	Thermal condition						Amplicon size (bp)	Reference
		Initial denaturation	Denaturation	Annealing	Elongation	Final elongation	Cycle		
*rcs*A	GGATATCTGACCAGTCGG GGGTTTTGCGTAATGATCTG	95°C, 5 min	95°C, 30 s	55°C, 30 s	72°C, 30 s	72°C, 10 min	30	109	(28)
*peh*X	GATACGGAGTATGCCTTTACGGTG TAGCCTTTATCAAGCGGATACTGG	95°C, 5 min	95°C, 1 min	55°C, 1 min	72°C, 1 min	72°C, 10 min	35	343	(29)
*fim*H	TGCTGCTGGGCTGGTCGATG GGGAGGGTGACGGTGACATC	94°C, 3 min	94°C, 45 s	55°C, 45 s	72°C, 45 s	72°C, 7 min	35	550	(30)
*uge*	GATCATCCGGTCTCCCTGTA TCTTCACGCCTTCCTTCACT	94°C, 5 min	94°C, 30 s	53°C, 30 s	72°C, 1 min	72°C, 10 min	35	534	
*ybt*S	GACGGAAACAGCACGGTAAA GAGCATAATAAGGCGAAAGA	95°C, 15 min	94°C, 30 s	60°C, 90 s	72°C, 60 s	72°C, 10 min	35	242	(31)
*mrk*D	AAGCTATCGCTGTACTTCCGGCA GGCGTTGGCGCTCAGATAGG							340	
*ent*B	GTCAACTGGGCCTTTGAGCCGTC TATGGGCGTAAACGCCGGTGAT							400	
*rmp*A	CATAAGAGTATTGGTTGACAG CTTGCATGAGCCATCTTTCA							416	
*kfu*	GGCCTTTGTCCAGAGCTACG GGGTCTGGCGCAGAGTATGC							638	
*all*S	CATTACGCACCTTTGTCAGC GAATGTGTCGGCGATCAGCTT							764	
*iut*A	GGGAAAGGCTTCTCTGCCAT TTATTCGCCACCACGCTCTT							920	
*mag*A	GGTGCTCTTTACATCATTGC GCAATGGCCATTTGCGTTAG							1,283	
K2	CAACCATGGTGGTCGATTAG TGGTAGCCATATCCCTTTGG							531	
*bla*_TEM_	ATAAAATTCTTGAAGACGAAA GACAGTTACAATGCTTAATCA	94°C, 5 min	94°C, 1 min	58°C, 1 min	72°C, 1 min	72°C, 8 min	35	1,080	(32)
*bla*_SHV_	GGGTTATTCTTATTTGTCGC TTAGCGTTGCCAGTGCTC	94°C, 4 min	94°C, 30 s	54°C, 30 s	72°C, 1 min	72°C, 10 min	34	915	(33)
*bla*_OXA-1_	ATATCTCTACTGTTGCATCTCC AAACCCTTCAAACCATCC	94°C, 4 min	94°C, 45 s	58°C, 1 min	72°C, 1 min	72°C, 5 min	32	619	(34)
*bla*_CTX-M_	ACGCTGTTGTTAGGAAGTG TTGAGGCTGGGTGAAGT	94°C, 5 min	94°C, 45 s	72°C, 45 s	72°C, 1 min	72°C, 5 min	35	759	(35)
*bla*_NDM_	GGTTTGGCGATCTGGTTTTC CGGAATGGCTCATCACGATC	94°C, 5 min	94°C, 30 s	58°C, 40 s	72°C, 50 s	72°C, 5 min	35	621	(36)
*bla*_OXA-48_	GCGTGGTTAAGGATGAACAC CATCAAGTTCAACCCAACCG	95°C, 5 min	95°C, 30 s	60°C, 1 min	72°C, 1 min	72°C, 7 min	35	438	
*bla*_KPC_	CGTCTAGTTCTGCTGTCTTG CTTGTCATCCTTGTTAGGCG	94°C, 3 min	94°C, 30 s	55°C, 30 s	72°C, 30 s	72°C, 7 min	30	798	

### Genomic DNA extraction and PCR amplification

Genomic DNA of all the isolates was extracted by boiling and throwing method based on Siddique et al. (37). DNA concentration and purity were assessed using a Nanodrop One (Thermo Fisher Scientific, USA). All PCRs were performed with a 25 µL reaction volume, each containing 1.0 U of Taq DNA polymerase (Takada Bio, Japan), 1.0 µM of each primer, 2.5 mM MgCl_2_, 0.2 mM of each dNTP, and 40 ng of genomic DNA template. The thermocycling conditions for PCR amplification of all primers are mentioned in Table 1. One multiplex PCR was conducted according to Compain et al. (31) to detect virulence genes. Amplified PCR products were then separated on a 1.5% agarose gel (Lonza SeaKem LE Agarose) in 1× TAE buffer (40 mM Tris-HCl, 1.18 mL acetic acid, 2 mM EDTA, pH 8.0), containing Safe Red DNA stain (Hebei Sanshi Bio-Tech Co. Ltd., China), at a constant voltage of 100 V for 30 min for electrophoresis, and the banding patterns were visualized and photographed using a Gel Doc 1000 documentation system (Bio-Rad Laboratories Inc., California, USA).

### Detection of virulence determinants and ESBL-carbapenemase-producing genes

All positive strains of both *K. pneumoniae* and *K. oxytoca* were subjected to a PCR assay to detect the existence of virulence genes (*ybt*S, *mrk*D, *fim*H, *kfu*, *rmp*A, *ent*B, *all*S, *iut*A, *uge*, and K2). ESBL-producing genes (*bla*_TEM_, *bla*_SHV_, *bla*_OXA-1_, *bla*_CTXm_) and carbapenemase-producing genes (*bla_N_*_DM_, *bla*_KPC_, and *bla*_OXA-48_) using previously reported primer sequences (Table 1).

### Biofilm formation assays (qualitative and quantitative)

To evaluate biofilm-forming ability, two complementary methods were used. First, the Congo red agar assay was performed according to Arciola et al. (38). Overnight cultures of isolated strains were streaked on Congo red agar (CRA) plates and incubated at 37°C for 48 h. Colony morphology was used to classify biofilm-forming capacities: dry filamentous crusty black colonies indicated strong biofilm; pink colonies with a dark center represented intermediate or potential biofilm producers, and smooth pink colonies were considered non-biofilm producers (39).

Secondly, a microtiter plate assay was conducted with slight modification of a previously described method by Coffey and Anderson (40). Overnight culture grown in LB broth was diluted in Trypticase soy broth (TSB) supplemented with 1% glucose and dispensed into a sterile 96-well microtiter plate (198 μL + 2 µL). Negative controls contained only sterile TSB. After incubation for 24 h at 37°C, non-adherent cells were removed by inversion and gently tapped onto absorbent paper. The wells were then washed four times with phosphate-buffered saline (PBS, pH 7.2). Biofilms were stained with 200 μL of 0.1% crystal violet for 15 min at room temperature, excess dye was removed by washing with distilled water, and bound dye was solubilized with 95% ethanol (10 to 15 min at room temp). Absorbance was measured at 550 nm using an ELISA plate reader (Thermo Fisher Scientific, USA), with wells containing only ethanol serving as blanks. Each assay was performed in duplicate to calculate the mean value. The cut-off optical density (ODc) for biofilm formation was defined as three standard deviations above the mean OD of the negative control. Based on OD value, isolates were categorized into non-, weak, moderate, or strong biofilm producers according to established criteria (41).

### Antibiotic susceptibility testing and multidrug-resistant analysis

Antibiotic susceptibility testing of all confirmed isolates was performed following Kirby-Bauer’s disc diffusion method in accordance with the Clinical and Laboratory Standards guidelines (42). Twenty commercially available antibiotics, commonly prescribed for UTI treatment, were used to determine the antibiotic susceptibility patterns of isolated strains. The β-lactam group included the penicillins (ampicillin [AMP; 10 μg], amoxicillin-clavulanic acid [AMC; 20–10 μg]), cephalosporins (ceftazidime [CZM; 30 μg], ceftriaxone [CRO; 30 μg], cefotaxime [CTX; 30 μg], cefepime [FEP; 30 μg]), monobactams (aztreonam [ATM; 30 μg]), carbapenems (imipenem [IMP; 10 μg], meropenem [MEP; 10 μg]), fluoroquinolones (ciprofloxacin [CIP; 5 μg], levofloxacin [LEV; 5 μg], norfloxacin [NOR; 10 μg], and nalidixic acid [NA; 30 μg]), aminoglycosides (gentamicin [CN; 10 μg], tobramycin [TOB; 10 μg], and amikacin [AK; 30 μg]), folate pathway inhibitors (sulfa drug) (trimethoprim-sulfamethoxazole [SXT; 25 μg] and cotrimoxazole [COT; 25 μg]), nitrofuran derivative (nitrofurantoin [F; 300 μg]), and the phosphonic acid derivatives (fosfomycin [FF; 30 μg]) (Oxoid, Germany). The MDR patterns were determined following the criteria of Magiorakos et al. (43), and the multiple antibiotic resistance (MAR) index was calculated as per Krumperman (44), defined as the ratio of the number of antibiotics to which an isolate exhibited resistance to the total number of antibiotics tested.

### Sequencing and phylogenetic analysis

Representative isolates from each confirmed *Klebsiella* species were selected for 16S rRNA gene sequencing to provide molecular species-level confirmation beyond biochemical identification and species-specific PCR, and to determine their phylogenetic placement relative to reference sequences deposited in NCBI GenBank. Genomic DNA from two isolates of *Klebsiella pneumoniae* (GenBank accession numbers PV821475 and PV821476) and two *K. oxytoca* isolates (GenBank accession numbers PX754938 and PX671591) was amplified by PCR using the primer sets (F: 5′-AGAGTTTGATCCTGGCTCAG-3′; R: 5′-CTTGTGCGGGCCCCCGTCAATTC-3′). Successful amplification was confirmed by agarose gel electrophoresis. The targeted amplicon was purified using the QIAamp PCR product purification kit (QIAgen GmbH, Hilden, Germany). Subsequently, the purified PCR products were sent to the BD Genomes Laboratory, Savar, Dhaka, for sequencing by the Sanger Dideoxy sequencing method. Consensus sequences were assembled using CLC Sequence Viewer v8 (http://www.clcbio.com) and queried against the NCBI GenBank database via BLAST.

For the construction of the phylogenetic tree, 16S ribosomal RNA of *Klebsiella pneumoniae* was downloaded from GenBank (https://www.ncbi.nlm.nih.gov/nucleotide/). After preliminary alignment with the study sequence, a total of 33 reference sequences were included for phylogenetic tree construction. The rest of the sequences were excluded due to shorter length. Maximum likelihood (ML) method with the best-fit model (TN93+G) was used to obtain evolutionary relationships, applying 1,000 bootstrap replications. Molecular Evolutionary Genetics Analysis version 11 (MEGA11) software was used for the tree construction. Initially, study and reference sequences were aligned using the MUSCLE algorithm present in MEGA11 software and exported as MEGA format. Finally, the tree was constructed using the above-mentioned model and annotated using the Interactive Tree Of Life v5: an online tool for phylogenetic tree display and annotation.

### Statistical analysis

All data were entered and analyzed using SPSS IBM software version 26.0. Descriptive statistics were calculated for demographic characteristics, clinical features, and prevalence of virulence and resistance genes. Given the small sample size (*n* = 22), Fisher’s exact test was used as the primary statistical test throughout to evaluate associations between virulence genes, antibiotic resistance genes, and biofilm formation categories. Chi-square tests were applied only where expected cell frequencies were ≥5 in all cells. Associations between virulence genes and biofilm formation were initially assessed using a Chi-square test or Fisher’s exact test, as appropriate. Crude odds ratios (ORs) and 95% confidence intervals (CIs) were calculated from 2 × 2 contingency tables. For the association between virulence gene presence and biofilm formation intensity, ordinal logistic regression analysis was performed using biofilm intensity category (non-producer, weak, moderate, and strong) as the ordinal dependent variable and virulence gene presence/absence as binary predictor variables. ORs and corresponding 95% CIs were estimated from the regression model coefficients. A *P* value < 0.05 was considered statistically significant. For continuous variables, such as optical density (OD) values from biofilm assays, Spearman’s correlation analysis was performed to assess the relationship with the MAR index. Statistical significance was considered at a *P* value < 0.05.

## RESULTS

### Demographic characteristics and prevalence of *K. pneumoniae*

**Fig 1:**
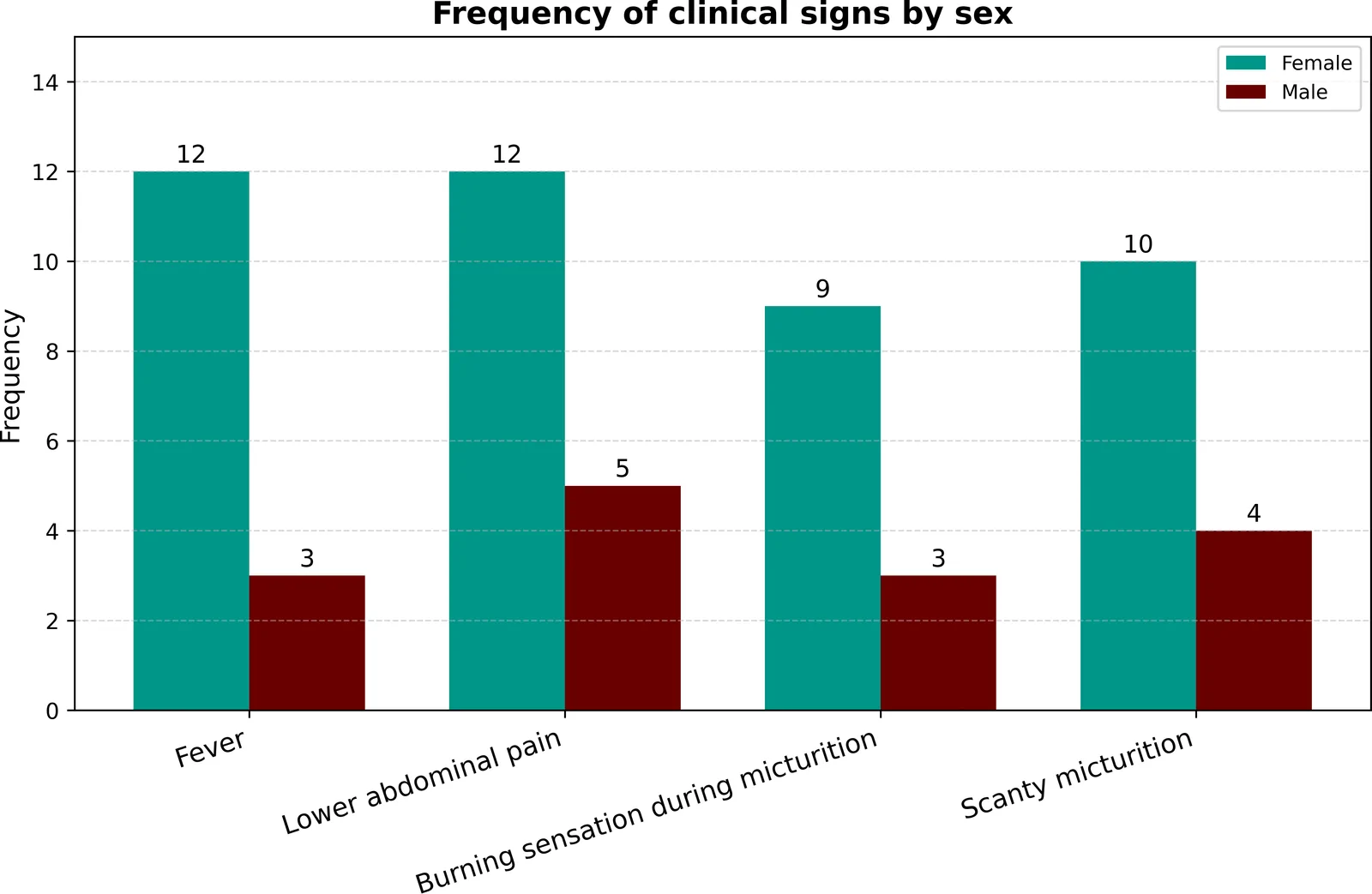
Frequency of clinical signs among male and female patients with *Klebsiella*-mediated UTI.

**Fig 2:**
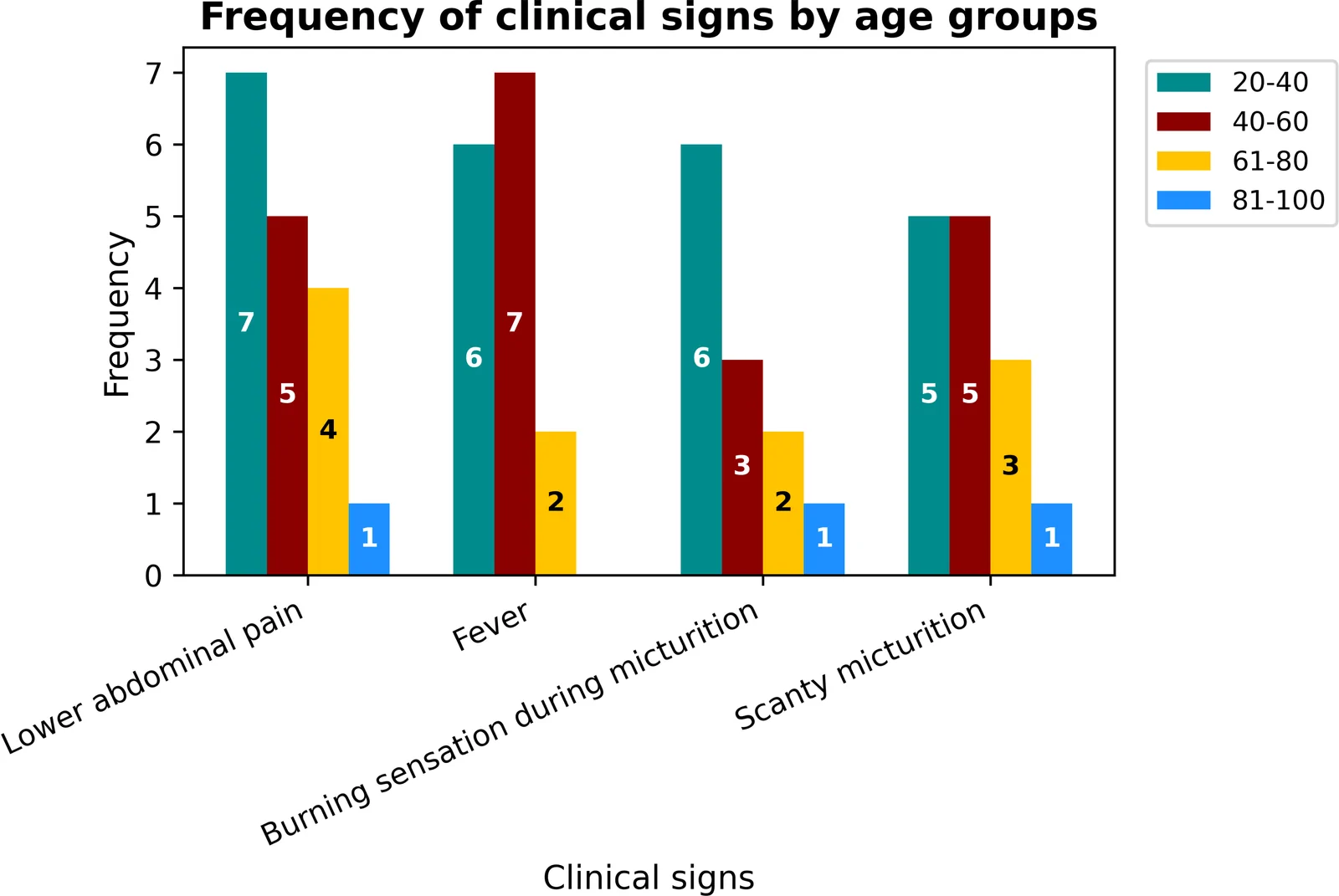
Age-wise distribution of clinical signs in *Klebsiella*-mediated UTI.

Among culture-positive isolates, 26 were initially suspected as *Klebsiella* spp. However, 22 isolates were confirmed as *Klebsiella* spp. by Gram’s staining, biochemical characterization, and PCR. Among them, 20 isolates were further identified as *K. pneumoniae* and 2 isolates as *K. oxytoca* by species-specific PCR (Fig. S1). Among the isolates, 77.3% (*n* = 17) were from female and 22.7% (*n* = 5) were from male patients. The highest prevalence was observed in the 20–40 years age group (45.5%), followed by 41–60 years (31.8%), 61–40 years (18.1%), and 81–100 years (4.5%). Clinical manifestations of symptoms like burning sensation during micturition (54.5%), fever (68.18%), and scanty micturition (63.6%); lower abdominal pain (77.27%) were recorded among infected patients; and their distribution according to sex and age group is presented in Fig. 1 and 2.

### Prevalence of virulence determinants

Among the investigated virulence determinants, *fim*H gene showed the highest prevalence, being detected in 90% (*n* = 18/20) of *K. pneumoniae* isolates and in one of the *K. oxytoca* isolates. This is followed by *mrk*D and *uge* genes, each present in 80% (*n* = 16/20) of the *K. pneumoniae* isolates, as well as in both *K. oxytoca* isolates. Moderate prevalence was observed for *ybt*S, *ent*B, *all*S, and *iut*A genes, which were identified in 30%, 30%, 35%, and 30% of *K. pneumoniae* isolates, respectively. Both *K. oxytoca* isolates contained only the *ybt*S gene. The lowest prevalence was recorded for *rmp*A and *kfu* genes, each detected in only 15% (*n* = 3/20) of *K. pneumoniae* isolates. None of the isolates carried the *mag*A gene or capsular serotype K2 (Fig. S2).

### Antimicrobial susceptibility profile, MAR index, and prevalence of MDR isolates

**Fig 3:**
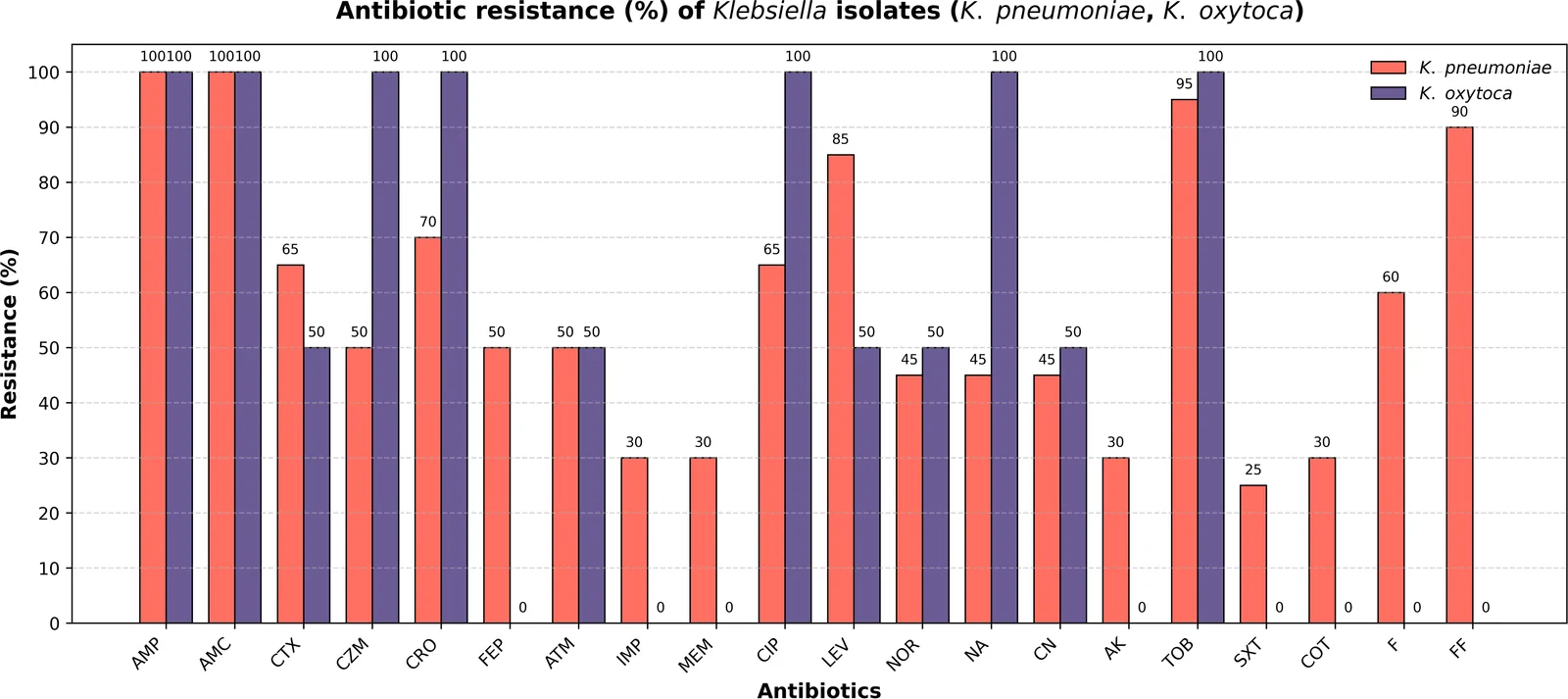
Antibiotic resistance profile (%) of *Klebsiella* isolates against tested antibiotics. AMP, ampicillin; AMC, amoxicillin-clavulanic acid; cephalosporins CZM, ceftazidime; CRO, ceftriaxone; CTX, cefotaxime; FEP, cefepime; ATM, aztreonam; IMP, imipenem; MEM, meropenem; CIP, ciprofloxacin; LEV, levofloxacin; NOR, norfloxacin; NA, nalidixic acid; CN, gentamicin; TOB, tobramycin; AK, amikacin; SXT, trimethoprim-sulfamethoxazole; COT, cotrimoxazole; F, nitrofurantoin; FF, fosfomycin.

All isolates showed 100% resistance to ampicillin and amoxicillin-clavulanic acid. Among cephalosporins, *K. pneumoniae* exhibited resistance rates of 50% to ceftazidime and cefepime, 65% to ceftriaxone, and 70% to cefotaxime, while both *K. oxytoca* strains were resistant to ceftazidime and ceftriaxone but susceptible to cefepime. About 70% of *K. pneumoniae* and both *K. oxytoca* were susceptible to carbapenems. Amikacin (70%) was the most effective aminoglycoside, whereas resistance to tobramycin was high. Fluoroquinolone resistance exceeds 60% for commonly used agents (ciprofloxacin, levofloxacin). In contrast, sulfonamide showed high efficacy (75% and 70% susceptibility) with both *K. oxytoca* being susceptible. High resistance to fosfomycin and nitrofurantoin was also observed in *K. pneumoniae,* while both *K. oxytoca* remained susceptible. The complete antibiogram susceptibility profile is presented in Fig. 3.

**Fig 4:**
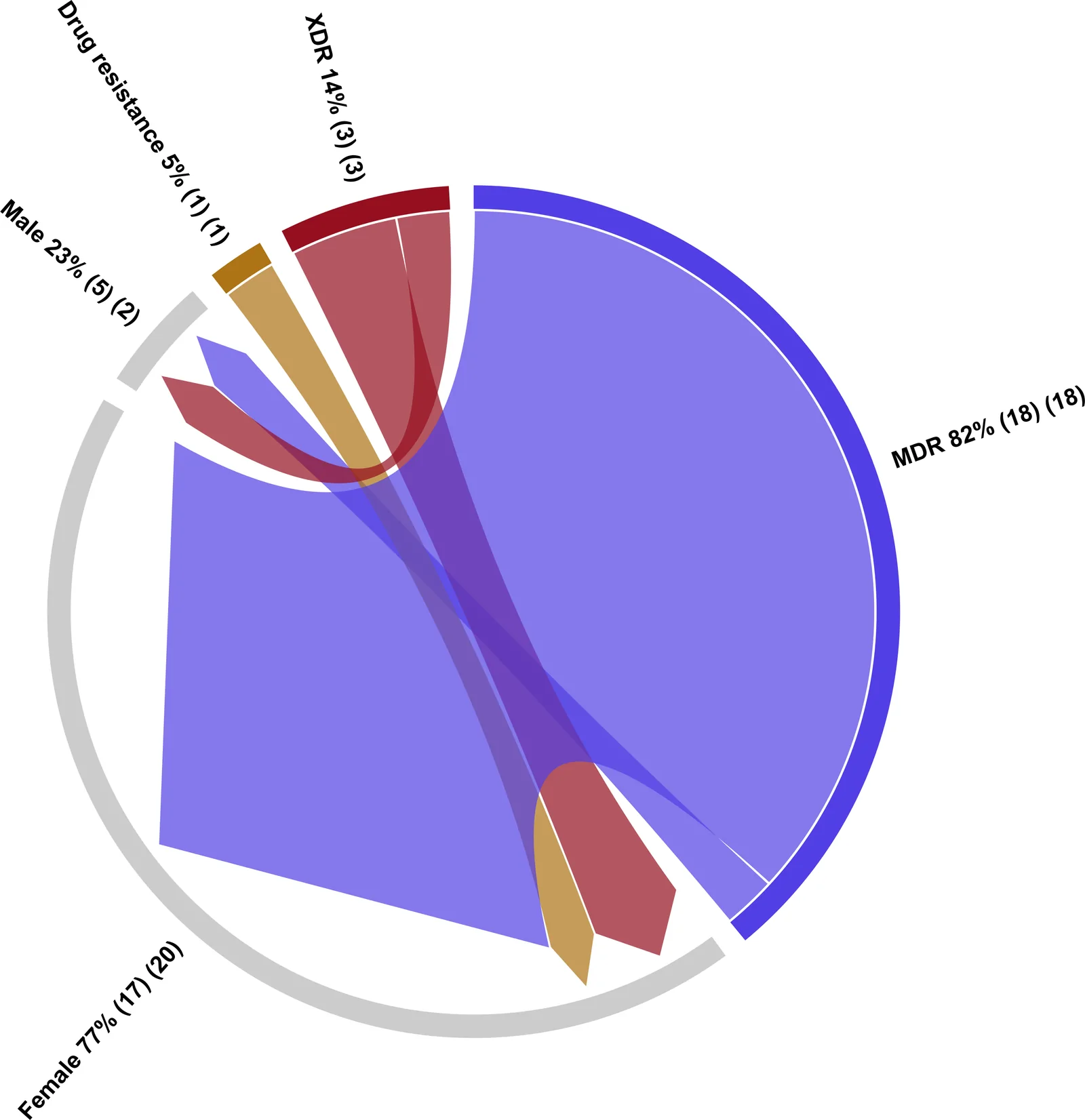
Chord diagram showing the relative distribution of antimicrobial resistance patterns across sex.

**Fig 5:**
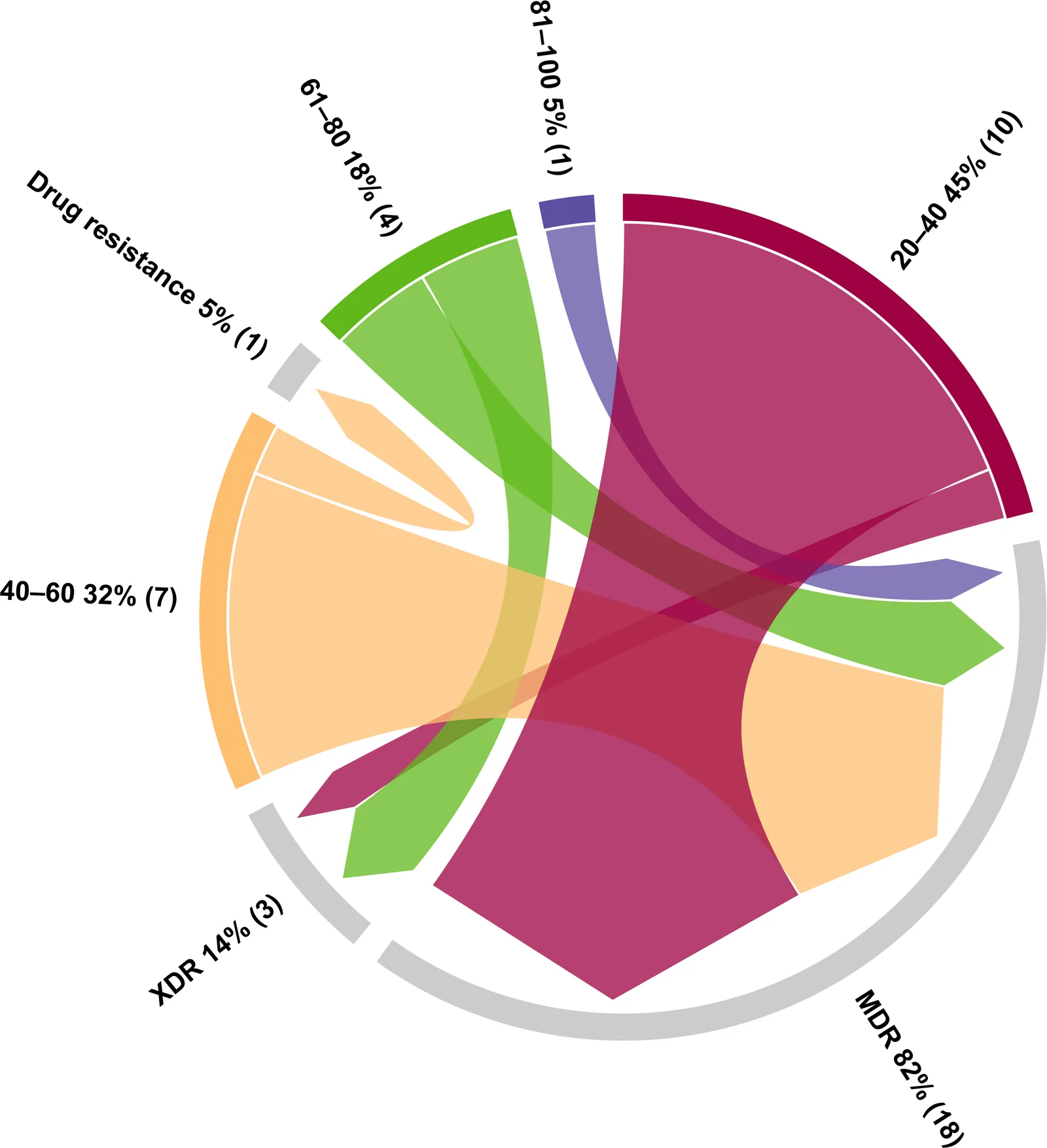
Distribution of antibiotic resistance patterns of *Klebsiella* isolates across different age groups.

The MAR index among all tested isolates ranged from 0.2 to 0.9. Analysis also showed that 95% (21/22) of the isolates exhibited an MDR resistance phenotype, resistance to three or more antibiotic classes. Whereas three *K. pneumoniae* strains can be considered as XDR (resistance to all tested antibiotic classes except one or two classes). However, no statistically significant association was observed between MDR/XDR status and patient sex (*P* = 0.783), age group (*P* = 0.250), or clinical signs (*P* = 0.552) (Table S1). Although MDR isolates were slightly more frequent among female patients (82.4%) compared to males (80.0%), and among the 40–609, 40–59 years age group, the difference was not significant (Fig. 4 and 5). Similarly, clinical symptoms, such as fever, scanty micturition, lower abdominal pain, and burning sensation with micturition, showed no significant relationship with the antimicrobial resistance pattern (*P* = 0.552).

### Prevalence of ESBL and carbapenemase-producing determinants

Among the 20 *K. pneumoniae* isolates, the most prevalent ESBL-encoding genes were *bla*_SHV_ (50%, *n* = 10) and *bla*_TEM_ (40%, *n* = 8), followed by *bla*_CTXm_ (30%, *n* = 6) and *bla*_OXA-1_ (25%, *n* = 5). Regarding carbapenemase genes, *bla*_NDM_ and *bla*_OXA-48_ were detected in 20% (*n* = 4) and 10% (*n* = 2) of *K. pneumoniae* isolates, respectively, whereas *bla*_KPC_ was not detected in any isolate. In contrast, among the two *K. oxytoca* isolates, only one harbored the *bla*_TEM_ gene, while all other resistance genes were absent (Fig. S3).

**Fig 6:**
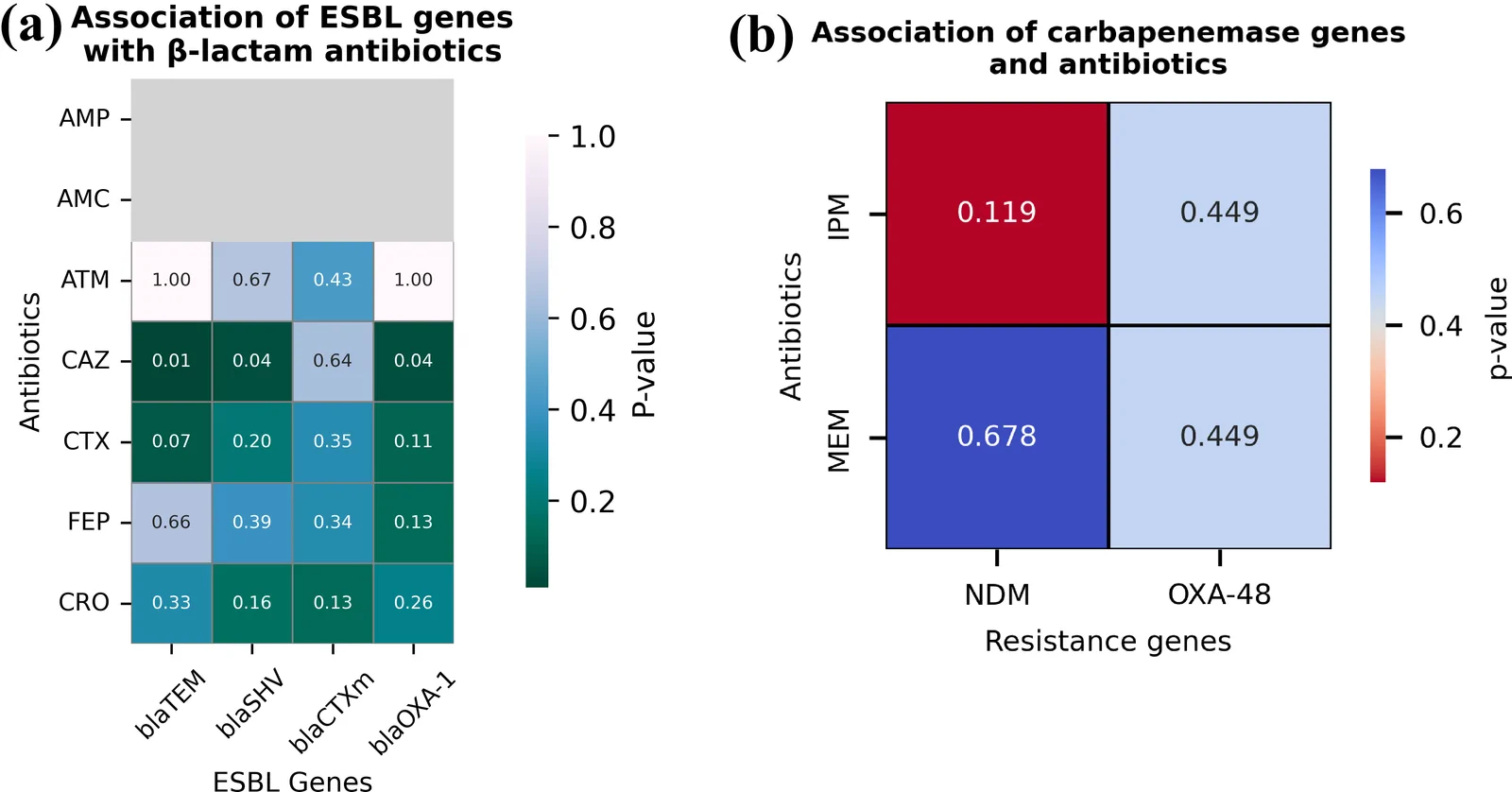
Heatmaps depicting correlations between ESBL genes and β-lactam antibiotics (**a**) and carbapenemase genes and carbapenems (**b**).

A significant link between β-lactam antibiotics and ESBL genes was observed. Specifically, resistance to ceftazidime was significantly tied to *bla*_TEM_, *bla*_SHV_, and *bla*_OXA-1_ (*P* < 0.05), but other antibiotics showed no significant associations. Likewise, there was no significant relationship between carbapenems and carbapenemase genes (Fig. 6).

### Biofilm-forming capability of the *Klebsiella* spp. isolates

Out of 22 tested isolates, phenotypic detection on CRA identified 13 (59.1%) as biofilm producers (5 strong and 8 intermediate), while 9 were non-biofilm producers. In contrast, the microtiter plate (MTP) assay revealed more detailed categorization of isolates as strong biofilm formers (18.2%, *n* = 4), moderate biofilm formers (27.3%, *n* = 6), weak biofilm formers (18.2%, *n* = 4), and non-biofilm formers (36.4%, *n* = 8).

### Statistical association between biofilm formation, phenotypic and genotypic resistance, and virulence determinants

Resistance was significantly associated with biofilm formation. Ampicillin and amoxicillin-clavulanic acid resistance was observed in all isolates across every category, so could not be tested statistically. Among the remaining antibiotics, significant associations between biofilm formation category and antibiotic resistance were observed for cefepime (*P* = 0.005), ciprofloxacin (*P* = 0.027), and nalidixic acid (*P* = 0.025) (Table 2). Overall, the resistance rate was higher in biofilm producers compared to non-biofilm producers. Moreover, a Spearman’s correlation analysis revealed a significant positive correlation (Spearman’s ρ = 0.537, *P* = 0.015) between biofilm OD value and the MAR index (Fig. S4).

**TABLE 2:** Association between biofilm formation strength and antibiotic resistance patterns of *Klebsiella pneumoniae* isolates^*a*^

Antibiotic	Non-biofilm	Weak	Moderate	Strong	χ² *P* value
Ampicillin	7	4	5	4	–^*b*^
Amoxicillin-clavulanic acid	7	4	5	4	–
Cefotaxime	3	3	4	3	0.273
Ceftazidime	1	3	3	3	0.123
Ceftriaxone	3	4	4	3	0.216
Cefepime	0	2	5	3	0.005
Aztreonam	2	2	4	2	0.379
Imipenem	1	2	1	2	0.460
Meropenem	2	2	0	2	0.299
Ciprofloxacin	3	1	5	4	0.027
Levofloxacin	5	3	5	4	0.405
Norfloxacin	2	2	3	2	0.729
Nalidixic acid	2	0	3	4	0.025
Gentamicin	5	1	1	4	0.075
Tobramycin	6	4	5	4	0.582
Amikacin	1	2	0	3	0.055
Trimethoprim-sulfamethoxazole	2	2	1	0	0.427
Cotrimoxazole	2	1	1	2	0.788
Nitrofurantoin	5	2	3	2	0.870
Fosfomycin	6	4	5	3	0.543

^*a*^ Resistance counts represent the number of resistant isolates per biofilm category. Pearson Chi-square test (df = 3). AMP and AMC were constant variables (all isolates resistant); statistical test not applicable. **P < 0.01; *P < 0.05; ns, not significant.

^*b*^ "–", No statistical comparison was possible because all biofilm-producing isolates were resistant.

Analysis of the association between virulence genes and biofilm formation revealed notable differences between unadjusted and adjusted results. In the univariate analysis (Chi-square test), only iutA and *fim*H were significantly associated with biofilm-positive isolates, suggesting a potential role in biofilm formation. However, when the effect of all genes was considered simultaneously, using ordinal regression, *mrk*D, iutA, and *all*S emerged as independent predictors of biofilm formation (Table 3).

**TABLE 3:** Association between virulence genes and biofilm formation among *Klebsiella pneumoniae* isolates^*a*^

Gene	Gene status	Biofilm absent/present (*n*)	Unadjusted OR (95% CI)	*P* value (Chi-square)	Adjusted OR (95% CI)	*P* value (ordinal logistic regression)
*ybt*S	Absent	4	0.240 (0.038–1.514)	0.119	24.35 (0.27–2,164.90)	0.163
	Present	3				
*mrk*D	Absent	2	1.571 (0.178–13.860)	0.683	0.012 (0.00024–0.655)	0.030
	Present	11				
*ent*B	Absent	8	5.000 (0.472–52.961)	0.157	10.30 (0.34–312.28)	0.180
	Present	5				
*rmp*A	Absent	9	1.300 (0.965–1.751)	0.121	20.13 (0.52–781.33)	0.108
	Present	3				
K2	Absent	0	–^*b*^	–	1	–
*kfu*	Absent	8	0.292 (0.022–3.830)	0.329	0.096 (0.00127–7.18)	0.287
	Present	1				
*all*S	Absent	7	2.188 (0.318–15.044)	0.421	0.013 (0.00027–0.619)	0.028
	Present	5				
*iut*A	Absent	7	1.857 (1.123–3.072)	0.017	0.0001 (0.0000–0.034)	0.002
	Present	6				
*fim*H	Absent	3	0.667 (0.420–1.058)	0.025	–	–
	Present	13				
*uge*	Absent	2	1.571 (0.178–13.860)	0.683	0.009 (0.00005–1.39)	0.067
	Present	11				
*mag*A	Absent	0	–	–	1	–

^*a*^ “OR,” odds ratio; “CI,” confidence interval; AOR, adjusted odds ratio; CI, confidence interval. Bold values indicate statistically significant associations (*P* < 0.05). Estimates were derived from a multivariable ordinal logistic regression model.

^*b*^ "–", No statistical comparison was possible because all biofilm-producing isolates were resistant.

**Fig 7:**
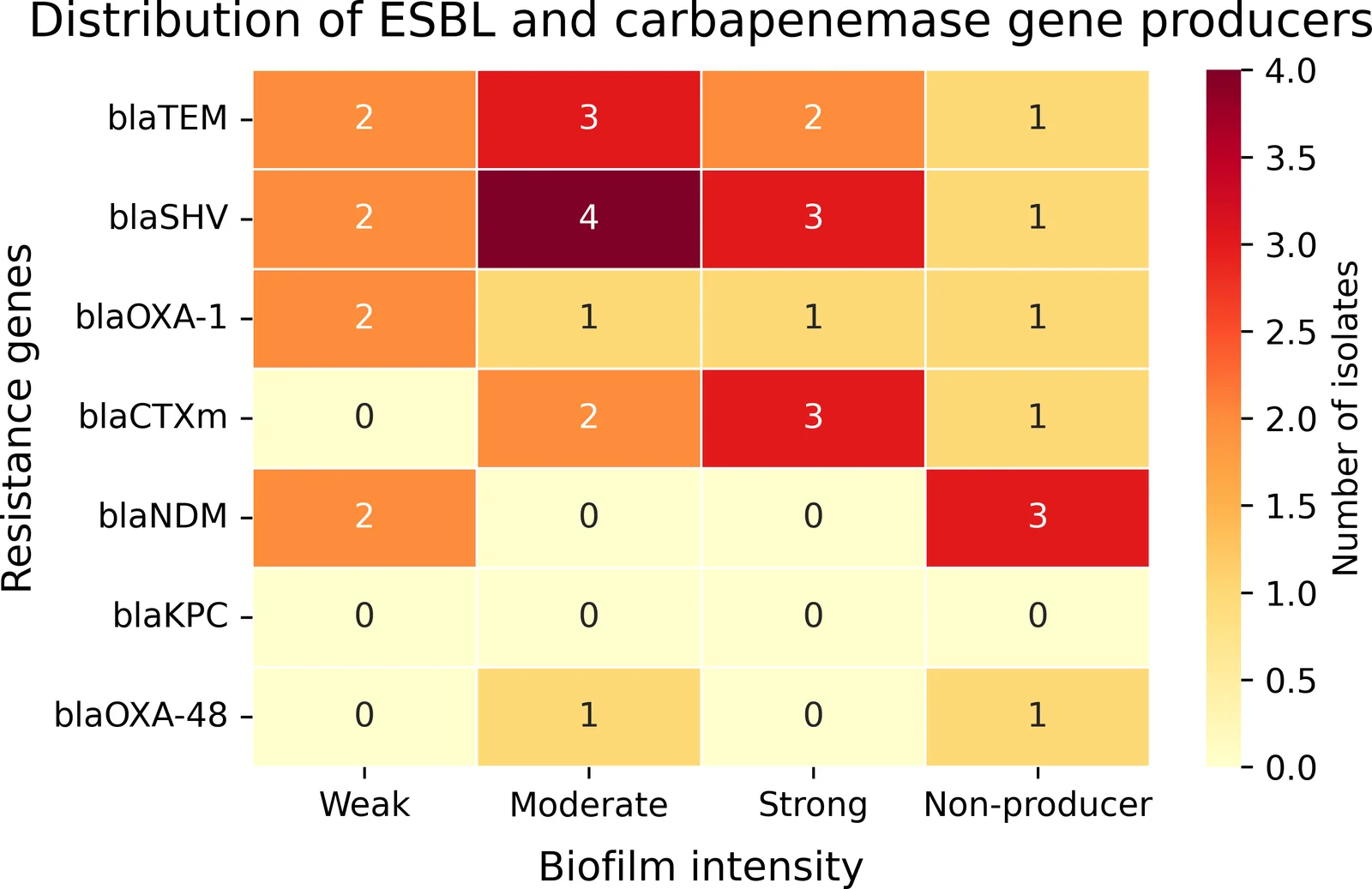
Relationship of ESBL and carbapenemase genes with biofilm-forming ability in *Klebsiella* isolates.

Distribution of β-lactamase genes differs significantly across the biofilm categories (*P* < 0.05). *bla*_TEM_ and *bla*_SHV_ were present in all categories, while *bla*_CTX-M_ was more common in the strong and moderate producers, and *bla*_NDM_ was reported in the weak and non-producers. Detailed distribution and significance are presented in Fig. 7.

### Sequence and phylogenetic analysis

**Fig 8:**
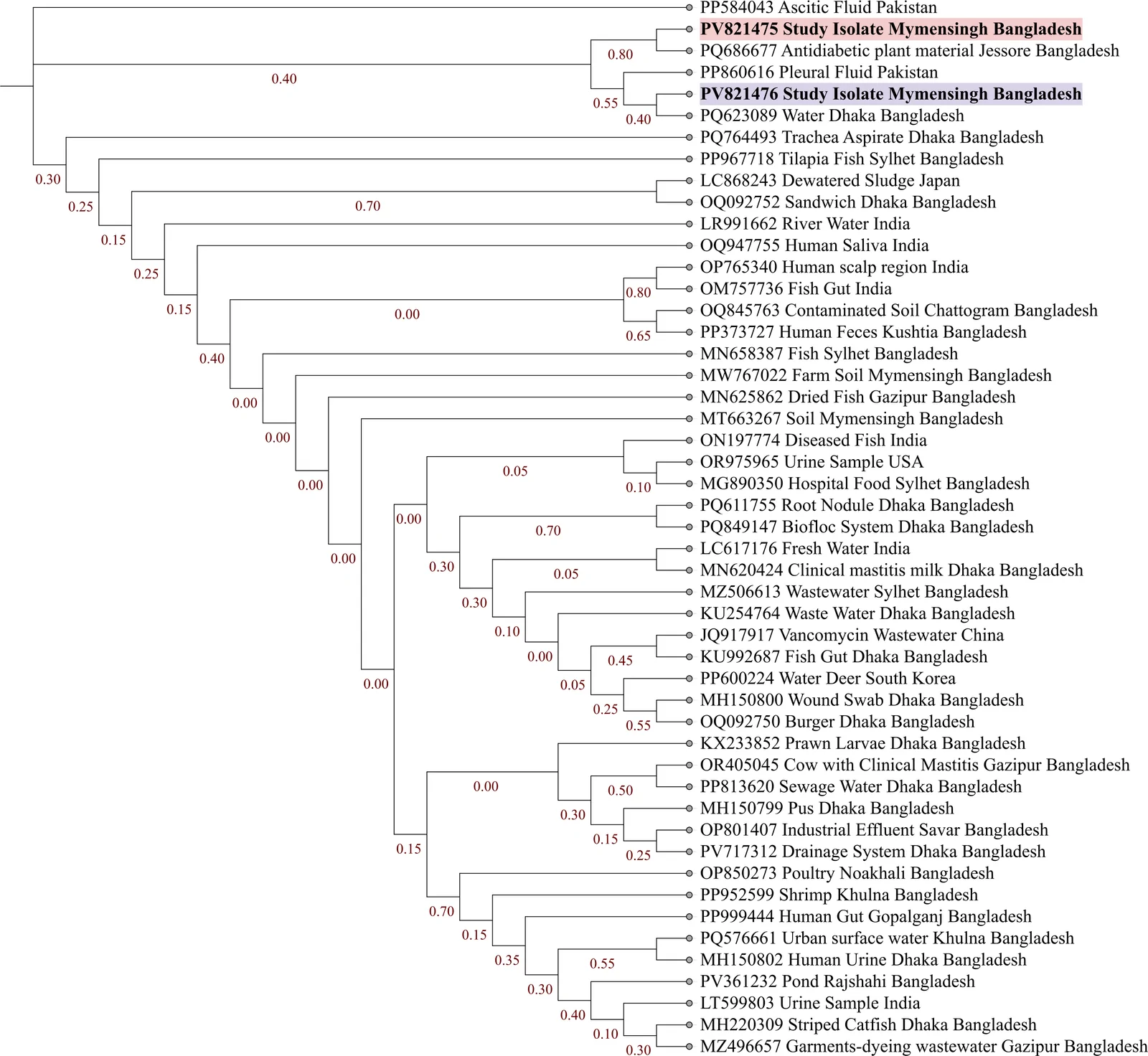
Phylogenetic tree showing the evolutionary relationship among *Klebsiella pneumoniae* study isolates and reference sequences from NCBI GenBank. Study isolates are indicated with colored bold text.

**Fig 9:**
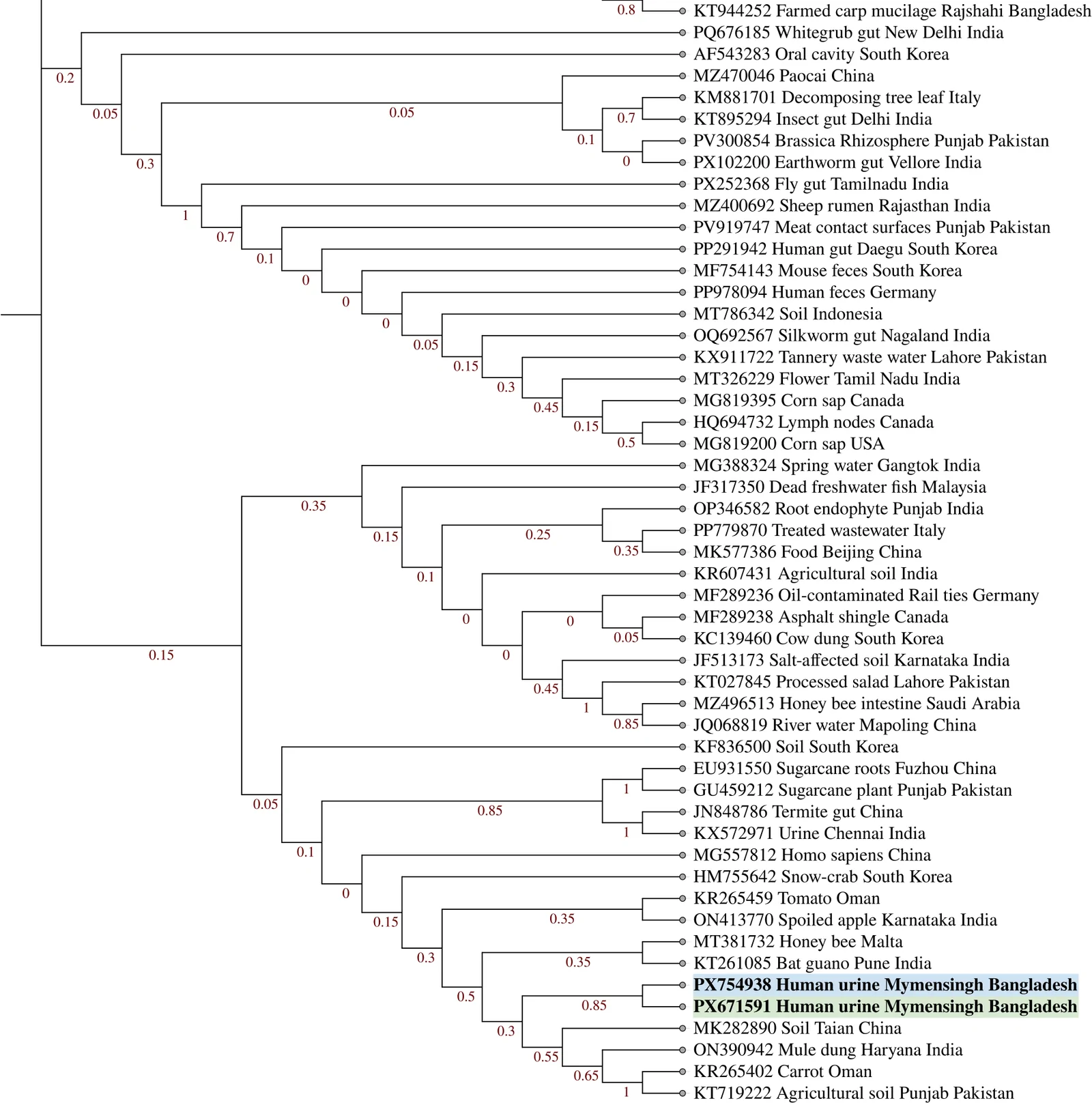
Phylogenetic tree showing the evolutionary relationship among *Klebsiella oxytoca* study isolates and reference sequences from NCBI GenBank. Study isolates are indicated with colored bold text.

A phylogenetic tree was constructed to determine the evolutionary relationship between the study isolates and the reference isolates already deposited in NCBI GenBank. In the analysis, the *Klebsiella pneumoniae* study isolates (PV821475 and PV821476) made a distinct cluster with reference strains, such as MN620424, PP373727, and KU992687, indicating their close genetic relatedness and suggesting a locally circulating lineage (Fig. 8). Similarly, the *K. oxytoca* isolates (PX671591 and PX754938) clustered within a well-supported clade containing references from Bangladesh, India, and Pakistan, reflecting close evolutionary relationships and possible regional circulation (Fig. 9). Clustering within the same clade indicates high genetic similarity between the study isolates and previously reported strains from neighboring regions.

## DISCUSSION

Among the members of the genus *Klebsiella* spp., *K. pneumoniae* and *K. oxytoca* have recently been recognized as the major cause of UTIs, next to *E. coli* (9, 45, 46). The role of these organisms in UTIs is particularly concerning due to their ability to persist in hospital environments, colonize indwelling medical devices, and thrive under antimicrobial pressure (6). The increasing prevalence of *Klebsiella* infections in hospital-acquired UTIs highlights its importance as a nosocomial pathogen. Previous studies have reported that *Klebsiella* species accounted for up to 20% of healthcare-associated infections (47, 48).

In this study, *K. pneumoniae* was isolated from 20 UTI cases, alongside two *K. oxytoca* strains. Supporting these findings, Eure et al. (6) also reported co-occurrence of *K. oxytoca* with *K. pneumoniae* in UTI patients. Notably, in this study, a predominance of isolates were recovered from female patients (77.3%, *n* = 17) compared with (22.7%, *n* = 5) from male patients. This observation is supported by earlier findings by Filve et al. (22), who reported a high frequency of UTIs caused by *K. pneumoniae* in females (63.6%, *n* = 7) compared to males (36.4%, *n* = 4). Our findings also align with previous studies in Bangladesh, which consistently identified *K. pneumoniae* as the second most common cause of UTIs in tertiary care hospitals. For instance, Islam et al. (18) documented a prevalence of 12.1%, second only to *E. coli* (51.6%), and further noted that women were disproportionately affected, with the majority (81%) of UTI patients being adults (>18 years), which also supports our report.

The pathogenicity of *Klebsiella* species in UTIs is strongly influenced by the presence of diverse virulence factors that mediate adhesion, biofilm formation, iron acquisition, capsule production, and immune evasion (13). In the present study, multiple virulence determinants were identified (*ybt*S, *mrk*D, *ent*B, *rmp*A, *all*S, *kfu*, *iut*A, K2, *mag*, *fim*H, *uge*), indicating the multifactorial strategies employed by this pathogen to establish infection. *fim*H was highly prevalent among 90% and *mrk*D was detected in 80% of *K. pneumoniae* isolates; no *mag* gene was identified. Interestingly, both *K. oxytoca* strains were also positive for the *mrk*D gene. These findings are consistent with those of Kot et al. (49), who reported 91% and 96% prevalence of *fim*H and *mrk*D genes in clinical isolates.

The capsule-associated gene *rmp*A was proposed as a biomarker for differentiating between hypervirulent (hvKp) and classical (cKp) *K. pneumoniae* strains (50), whereas the K2 gene, related to capsular serotype, has been implicated in severe invasive infections, especially those associated with liver abscesses and sepsis (51). In our study, three strains (15%) of *K. pneumoniae* carried the *rmp*A gene, while none harbored the K2 serotype. Similarly, Ballén et al. (17) reported no prevalence of the K2 serotype in urinary isolates. The *uge* (uridine diphosphate galacturonate-4 epimerase) gene, which contributes to capsule formation, lipopolysaccharide synthesis, and serum resistance, was identified in 80% of isolates. Kot et al. (49) similarly reported nine hyper-viscous isolates containing the *rmp*A gene and the presence of the *uge* gene in the majority of urinary isolates, emphasizing its role in UTI pathogenesis. Mutations in the *uge* gene reduce the colonization efficacy of *K. pneumoniae* in experimental UTIs (52, 53). However, the prevalence of the *uge* gene in *K. pneumoniae* varies considerably (48.6% to 84.2%) across studies (17, 52).

Siderophore production is another virulent mechanism of *Klebsiella* species. Kot et al. (49) reported 100% prevalence of *ent*B gene encoding enterobactin in clinical isolates of *K. pneumoniae*, whereas in our study, the prevalence of *ent*B gene was markedly lower (30%). However, this outcome is consistent with the observation that Shanmathy et al. (54) reported a 37.6% prevalence of *entB* gene in uropathogenic *K. pneumoniae*. The *iut*A gene, encoding the aerobactin receptor, a potential siderophore commonly associated with hypervirulence, was present in 30% (*n* = 6/20) of *K. pneumoniae* strains. The *ybt*S gene, part of the yersiniabactin system, was found in 30% of the *K. pneumoniae* isolates and in both *K. oxytoca* strains. The kfu gene is detected more frequently in invasive clinical isolates; particularly those derived from liver abscesses and meningitis cases, compared to noninvasive strains (11). This study detected kfu in 15% of *K. pneumoniae* isolates. Moreover, the prevalence of *all*S gene was found to be 35% among the isolates, responsible for allantoin metabolism, enhancing persistence in extraintestinal sites (48). These findings align with those of Davoudabadi et al. (7), who reported carriage of virulence factor genes, including *iuc*B (24%), *iut*A (46.2%), *iro*N (25%), *kfu* (11%), *all*S (17.3%), from clinical samples.

While virulence factors contribute to pathogenicity, the therapeutic challenge posed by *Klebsiella* is largely dictated by their antibiotic resistance profile. Recent studies reported that the antibiotic resistance in *K. pneumoniae* has progressed from MDR to XDR and PDR (55, 56). In this study, 95% of *K. pneumoniae* isolates (19/20) exhibited an MDR phenotype, and both *K. oxytoca* isolates (2/2) were also MDR, giving an overall MDR rate of 95.5% (21/22) across the collection. In addition, three isolates were classified as XDR, retaining susceptibility to only a limited number of antibiotics (nitrofurantoin, aztreonam, and sulfonamides), which likely reflects strong selective pressure due to repeated and inappropriate antimicrobial exposure. Besides, the calculated MAR index ranged from 0.2 to 0.9, indicating a high risk of treatment failure and extensive exposure of these isolates to antimicrobials. The susceptibility pattern revealed complete resistance to penicillins and largely resistance to cephalosporins. Carbapenems are often considered the drug of last resort for complicated UTIs; they remain effective in most strains, possibly reflecting their relatively restricted use. Nitrofurantoin, trimethoprim-sulfamethoxazole (TMP-SMX), and fosfomycin are the first-line choices of drugs to treat UTIs. Therefore, isolates exhibited >70% sensitivity to TMP-SMX, whereas resistance to fosfomycin and nitrofurantoin was high, with *K. oxytoca* remaining fully susceptible. Fluoroquinolones and aminoglycosides are often considered as the second-line choices in UTI therapy, demonstrating heterogeneous resistance profiles in our isolates. Amikacin was most effective, whereas 45% of *K. pneumoniae* isolates were resistant to gentamicin. Both *K. oxytoca* strains were sensitive to amikacin, and one remained susceptible to gentamicin. These findings are largely consistent with previous reports from Bangladesh and other countries. Islam et al. (18) reported a high MDR rate among uropathogenic *Klebsiella* spp. (68%), with resistant penicillin (85%–95%), macrolide (70%–76%), third-generation cephalosporins (69%–58%), fluoroquinolone (69%–53%), and sulfonamide (56%–41%), whereas carbapenem resistance was relatively low (9%). Similarly, Chowdhury et al. (2) observed 48.59% MDR uropathogenic *K. pneumoniae*, with notable resistance (40%) to TMP-SMX, cefuroxime, and ceftazidime, while amikacin remained highly effective (only 9.6% resistance). Davoudabadi et al. (7) also reported MDR, XDR, and PDR rates of 84.6%, 13.5%, and 1.9%, respectively, in clinical *K. pneumoniae* isolates.

The clinical impact of *K. pneumoniae* is further aggravated by its remarkable ability to acquire and disseminate resistance determinants, particularly against β-lactam antibiotics. These antibiotics target PBPs to disrupt cell wall synthesis (55). According to the Ambler classification, β-lactamases are divided into four classes (A–D). Class A enzymes include KPC, ESBLs, TEM-1, SHV-1, and CTX-M, which confer resistance to penicillins and cephalosporins; class B (NDM, VIM, IMP) enzymes hydrolyze a wide range of β-lactam antibiotics, including carbapenems. Class C (AmpC) targets cephamycins and third-generation cephalosporins; class D (OXA-type) resists oxacillin and some carbapenems (55, 57). In our study, *bla*_TEM_ and *bla*_SHV_ were most prevalent (40% and 50%), and *bla*_CTX-M_ was observed in 30% of the *K. pneumoniae* isolates, consistent with the report of Müller-Schulte et al. (58), who also observed significant prevalence of ESBL genes (TEM, SHV, and CTX-M) among *K. pneumoniae* strains from UTIs. Additionally, OXA-1 was detected in 25% (*n* = 6) of our *K. pneumoniae* isolates, which is higher than the 10% prevalence reported by Lau et al. (59) in the clinical *K. pneumoniae* strains. Carbapenems represent a unique subclass of β-lactam with broad spectrum and potential antibacterial activity (55). Resistance to these agents in *Klebsiella pneumoniae* is primarily mediated by the production of carbapenemase, such as *K. pneumoniae* carbapenemase (KPC). In the present study, however, *bla*_KPC_ was not detected in any isolates. Instead, *bla*_NDM_ and *bla*_OXA-48_ were identified in 20% (*n* = 4) and 10% (*n* = 2) of *K. pneumoniae* isolates, respectively. Consistent with our findings, Davoudabadi et al. (7) detected *bla*_OXA-48_ gene in (9.6%, *n* = 5) and *bla*_NDM_ in (11.5%, *n* = 6) *K. pneumoniae* isolates, whereas *bla*_KPC_, *bla*_IMP_, and *bla*_VIM_ were absent.

The pathogenic success of *Klebsiella* is further reinforced by its strong biofilm-forming ability. Biofilm protects bacteria from environmental stress, increases resistance compared to planktonic counterparts (24). In our study, 59.1% of isolates demonstrated biofilm-forming capacity, with 18.1% strong, 27.3% moderate, and 18.1% weak biofilm producers. These findings are slightly lower than those reported by Hamam et al. (60), who observed 81% prevalence of biofilm-forming *K. pneumoniae* from UTIs. However, our results are consistent with Karimi et al. (61), who reported biofilm formation in 74.5% of *K. pneumoniae* isolates, with 32.5%, 21.6%, and 20.4% of weak, moderate, and strong biofilm producers, respectively. The variation in biofilm prevalence in this study compared with previous reports may be attributed to differences in virulence genes or plasmid content and study conditions. Notably, MTP assay was emphasized in this study as it provides a quantitative, objective, and more sensitive assessment of surface-associated biofilm biomass compared with the qualitative and visually interpreted Congo red agar method (62).

Biofilm is not a result of a single gene but rather the combined action of adhesion, capsule, and iron acquisition genes. *mrk*D, *mrk*A, and *fim*H genes generally showed moderate to strong biofilm production. Hypervirulent genes (*rmp*A and *mag*A) increase capsule thickness and strengthen biofilm (13). Given that biofilm creates an iron-limited microenvironment, iron acquisition genes (*ybt*S, *kfu*) are associated with strong biofilm production (63). In this study, univariate analysis (Chi-square) identified iutA (*P* = 0.017) and *fim*H (*P* = 0.025) as significantly associated with biofilm-positive isolates. However, when adjusted for multiple virulence factors using ordinal regression, mrkD, allS, and *iut*A emerged as independent predictors of biofilm formation. A previous study similarly reported a strong association of *mrk*D and fimH genes with biofilm-producing strains (64). These results highlight that multiple variable determinants act synergistically in biofilm formation, which in turn facilitates persistent infection treatment failure and MDR dissemination.

Additionally, biofilm has been reported to exhibit 10–1,000 times greater tolerance to antimicrobial agents compared with planktonic cells, which may contribute to the development of MDR (16). In this study, a significant association was observed between biofilm formation and antibiotic resistance phenotype for cefepime (*P* = 0.005), ciprofloxacin (*P* = 0.027), and nalidixic acid (*P* = 0.025), with strong and moderate producers exhibiting higher resistance rates compared to weak and non-biofilm producers. Gentamicin (*P* = 0.073) and amikacin (*P* = 0.055) showed borderline associations. These findings align with Hamam et al. (60), who reported a significant association between biofilm production and resistance to amikacin, cefepime, ceftazidime, gentamicin, imipenem, and meropenem of *K. pneumoniae*. A positive significant correlation was noted between biofilm OD value and MAR index (Spearman’s ρ = 0.537, *P* = 0.015). Similar findings were reported by Karimi et al. (61), who demonstrated a significant correlation between biofilm formation and resistance to multiple antibiotics (*P* value < 0.05), and by Sayed et al. (8), who identified a significant association between MDR and biofilm formation among *K. pneumoniae* and *E. coli* isolates (*P* = 0.016 and *P* = 0.001, respectively). Such a correlation highlights the potential role of biofilm formation in the persistence and spread of antibiotic resistance, particularly in healthcare settings.

Recent studies demonstrate a strong link between biofilm formation and horizontal gene transfer. Conjugation and transduction occurred more efficiently in biofilm due to close cell proximity and nucleic acid retention within the extracellular polymeric substance matrix (65). Consistent with this mechanism, our study revealed a significant distribution of resistant determinants across the biofilm categories (*P* < 0.05). The majority of biofilm-producing strains carried beta-lactamase determinants. While *bla*_TEM_ and *bla*_SHV_ were present in all categories, *bla*_CTX-M_ was predominantly detected in the strong and moderate producers; whereas *bla*_NDM_ was frequently identified in the weak and non-producers. These results support the previous findings of Hamam et al. (60), which reported a higher prevalence of ESBL determinants, particularly *bla*_SHV_ and *bla*_CTX-M_, among biofilm-forming strains.

High prevalence of multidrug resistance coupled with the detection of critical ESBL and carbapenemase genes highlights the growing therapeutic challenge posed by *Klebsiella* species, especially *K. pneumoniae,* in the clinical practice of UTI. Furthermore, the observed association of biofilm with antibiotic resistance revealed that biofilm is a key driver for persistent infection and treatment failure. However, certain limitations should be acknowledged. First, the relatively small sample size limits the statistical power for group comparison. In particular, findings related to *K. oxytoca* should be treated as entirely descriptive; no inferential conclusions can be drawn regarding that species, as they are based only on two isolates. Second, the study’s restriction to a single tertiary health care setting in Mymensingh limits generalizability. Third, the antibiotic exclusion criterion of 3 days prior to sample collection may be insufficient to completely eliminate the recent antimicrobial exposure on the urinary microbiota. As a result, the background result of resistance prevalence may be overestimated. Future studies should consider using a longer exclusion period of at least 14 days. Fourth, a limitation of the virulence gene PCR screening is the absence of certified positive control reference strains; while expected amplicon sizes were used to confirm amplification fidelity, formal validation against characterized ATCC strains would strengthen the reliability of these findings. Moreover, the lack of whole-genome sequencing to fully characterize resistance determinants and the absence of functional assays to validate virulence phenotype is limited in this study. Future studies with larger, multi-center sample collections are essential to validate the patterns observed here.

### Conclusion

In conclusion, this study demonstrated that *K. pneumoniae* and *K. oxytoca* isolates from urinary tract infections exhibit a high prevalence of virulence genes, multidrug resistance, and biofilm-forming ability, factors that collectively enhance their pathogenic potential and complicate clinical management. The detection of ESBL and carbapenemase genes, along with biofilm production, highlights urgent needs for continuous surveillance, antimicrobial stewardship, and the development of alternative treatment strategies to mitigate the impact of these opportunistic pathogens.

## Data Availability

All data generated or analyzed during this study are included in this manuscript and its supplemental materials.

## SUPPLEMENTAL MATERIAL

Fig. S1 spectrum.00664-26-s0001.tif Species-specific PCR identification of *Klebsiella pneumoniae* and *Klebsiella oxytoca*.

Fig. S2 spectrum.00664-26-s0002.tif Gel electrophoresis showing the presence of virulence genes in *Klebsiella* isolates.

Fig. S3 spectrum.00664-26-s0003.tif Amplification of ESBL- and carbapenemase-encoding genes in *Klebsiella* isolates.

Fig. S4 spectrum.00664-26-s0004.tif Correlation between biofilm formation and MAR index.

Table S1 spectrum.00664-26-s0005.docx Association between demographic characteristics, clinical signs, and multidrug resistance (MDR/XDR) in *Klebsiella pneumoniae* isolates.

ASM does not own the copyrights to Supplemental Material that may be linked to, or accessed through, an article. The authors have granted ASM a non-exclusive, world-wide license to publish the Supplemental Material files. Please contact the corresponding author directly for reuse.
