# Supplementary material for: Biofilm, virulence, and ESBL-mediated resistance: unmasking Klebsiella spp. UTIs
Source: Microbiol Spectr. 2026 Jul 27;14(9):e00664-26. doi: 10.1128/spectrum.00664-26 (PMC13532184; doi:10.1128/spectrum.00664-26)
Supplement: Supplementary file 1 [file spectrum.00664-26-s0005.docx]

### Table S1: Association between demographic characteristics, clinical signs, and multidrug resistance (MDR/XDR) in *Klebsiella pneumoniae* isolates

| **Demographic patient data** | **MDR (n, %)** | **XDR (n, %)** | **Drug resistance (n, %)** | **P-value** |
| --- | --- | --- | --- | --- |
| **Sex** |  |  |  | 0.783 |
| Male | 4 (80.0%) | 1 (20.0%) | 0 (0.0%) |  |
| Female | 14 (82.4%) | 2 (11.8%) | 1 (5.9%) |  |
| **Age group (years)** |  |  |  | 0.250 |
| 20–40 | 9 (40.9%) | 1 (4.5%) | 0 (0.0%) |  |
| 41–60 | 6 (27.3%) | 0 (0.0%) | 1 (4.5%) |  |
| 61–80 | 2 (9.1%) | 2 (9.1%) | 0 (0.0%) |  |
| >80 | 1 (4.5%) | 0 (0.0%) | 0 (0.0%) |  |
| **Clinical signs** |  |  |  | 0.717 |
| Fever | 5 (22.7%) | 1 (4.5%) | 0 (0.0%) |  |
| Scanty micturition | 4 (18.2%) | 1 (4.5%) | 0 (0.0%) |  |
| Lower abdominal pain | 6 (27.3%) | 0 (0.0%) | 1 (4.5%) |  |
| Burning sensation with micturition | 3 (13.6%) | 1 (4.5%) | 0 (0.0%) |  |

**Note:** “MDR” Multi drug resistance; “XDR” Extreme drug resistance. P-values calculated using Chi-square or Fisher’s exact test, as appropriate, to assess associations between demographic/clinical variables and resistance categories. All comparisons were non-significant (p > 0.05), indicating no significant association between demographic or clinical characteristics and antimicrobial resistance category.
